# Nature's Methods of Defense

**Published:** 1899-03

**Authors:** L. P. Bethel

**Affiliations:** Kent, O.


ARTICLE II.

### NATURE'S METHODS OF DEFENSE.

L. P. BETHEL, KENT, O.

Throughout the life of all organic bodies there is constant warfare; foe attacks foe; a continual battle for existence.

Man, animals and other complex organic bodies are made of a multiplicity of cells; millions upon millions of these minute particles of living matter uniting to form the whole. Each cell has a life of its own to live, and when it dies it is removed and another is ready to take its place. Thus organic bodies are ever changing. One organ of the body seems constantly trying to outdo some other organ, and, indeed, it would seem that even among cells of the same kind there is not mutual aid, but perpetual strife. Every part that increases, determines the enfeeblement of other parts. The stronger cell attacks the older and weaker cells, and, like the Hottentots, kill off the parents when their usefulness is spent.

Bichat said, "Life is the sum of the functions which resist death." We might represent it as being made up of two principal forces: First, that of building up the organism during the period of growth, and keeping up the repair during middle life and declining years. Second, a resisting force that stands guard ever watchful to keep the system in order, and always ready to resist the attacks of invaders from without.

We have referred only incidentally to the first, for the second is the one about which we desire more particularly to speak. Every growth has its foes, and the higher we go in the scale of life the more there is with which the organism has to contend. The resistive force, however, is stronger in some species than in others. Certain plants and trees exhale particular odors or excrete juices that are antiseptic and destroy the parasites that attack them. Although this resistive force is present in all forms of organic life, it is complete in animals and man.

Man is provided with muscular strength to defend himself against visible things, but it is through the wise provisions of nature that he is enabled to withstand the attacks of invisible foes, such as pathogenic or disease-producing germs

It is interesting to trace the methods of defense with which nature has endowed man, and by way of illustration we will pre-suppose the presence of disease-producing bacteria seeking entrance to the human body. Let it first be understood, however, that bacteria are vegetable organisms and not animal. They do not fly nor run; they simply grow on favorable soil as a plant would grow.

Being so minute, every disturbance of air will lift them into the atmosphere, and they find lodgment on everything. The dust of our streets is laden with germs, and they abound in profusion in places where sanitary conditions are not good. This points out the necessity for good ventilation, keeping our apartments well aired and bathed in sunshine, for sunlight is one of the best of disinfectants. Bacteria finds lodgment on our clothes; skin, hair, and we are constantly breathing them. Researches of Professor Thompson showed that under the most favorable conditions, the lowest number of organisms contained in the inhaled air of an hour, was fifteen hundred; and that in a large city the air that passed through the nose in the same length of time was charged with from fifteen to sixteen thousand. Many times even a greater number are present. The fate of the thousands of microbes which thus enters the human body is a question of great pathologic interest, and this increases when it is remembered that expired air is practically free from germs. Fortunately they are not all pathogenic organisms, but they are all invisible to the naked eye; and the pathogenic are intermingled with the non-pathogenic germs, so man cannot select air that is free from them. Here nature steps to the rescue and provides first a nose that is turning downward. Bacteria being heavier than air, they are constantly falling, and in consequence fewer are inhaled than would be the case if the nose turned upward and served as a "catch all." But this is not the only precaution against their invasion. When bacteria are inhaled they meet resistive force in the nostrils. First, the vibrissæ, or hairs in the nose, arrest many of the inhaled germs Others are caught by the mucus and carried o t of the nose. The nasal mucus being somewhat antiseptic; the surface it covers is rendered non-suitable for the growth of germs. Then, the cilia of the epithelium are active in rejecting the invaders. We might say, then, that the nose acts as a germ and dust filter, arresting these minute particles before they reach the trachea or bronchi.

The mouth breather runs more risk, for the inhaled germs are carried immediately to various parts of the mouth and throat, the pharyngial wall and tonsils being favorite locations for development and growth of diphtheria-producing and some other bacteria. Hence the dentist should breathe through his nose, especially when burning out tooth cavities and inhaling the dust.

When we disobey the laws of nature, we take the risk in our own hands. "Keep your mouth shut and save your life," is prudent advice. Yet, in the healthy individual the germs inhaled or taken into the mouth with food and water, meet immediate opposition. The saliva and mucus in the mouth and throat catch them, and they are carried into the stomach to be put to death by the gastric secretions. If any survive this, they are carried by the cilia along the intestinal tract and are eliminated. Should they lodge in the bronchi, they are arrested by the secretions and borne away by the cilia.

The saliva and mucus, in normal condition, are somewhat antiseptic, and prevent development of many germs. Yet some do thrive in the mouth, but so long as the tissues are normal, their secreted juices protect them from bacterial attacks. Should bacteria attempt entrance by way of the ear, they are arrested by the epidermis and the secreted wax. If by the eyes, the secretions carry them away. If they are on the skin, they find a barrier in the cell of the epidermis, for there is a continual scaling off of this membrane, and adherent bacteria are carried with the scales. Then there are the glands of the skin, throwing out sweat and oily materials that arrest or carry away the bacteria, even though they succeed in penetrating the glands. Here is a plea for cleanliness; for when we bathe, this army of microbes are washed away and the glands are freed from extraneous matter clogging their mouths, and stimulated to renew action.

Sometimes bacteria get to the internal tissues through a hair follicle or minute opening in the skin, but still all is not overcome for internal resistence is here encountered. The juices of the body are highly antiseptic, and promptly kill or weaken the vitality of the microbes. Now, suppose they withstand all this and succeed in getting into the blood circulation, still they find opposition. In the blood are wandering cells, white blood corpuscles, or phagocytes, that, like some policemen, keep vigilant watch, and appear on the spot when the invaders break in, and attempt their im ediate arrest. These phagocytes are not so numerous as red blood corpuscles, the red predominating in the proportion of four hundred to one. But when the internal tissues are threatened, the white corpuscles rapidly increase in numbers. They differ from the disk-like red corpusles, being composed of a plastic protoplasm, which enables them to assume various shapes, and to slip into the smallest intercellular spaces. When a bacterial invader reaches the interior of a blood-vessel, it is immediately approached by one of these wandering cells. Phagocytes being of various shapes, it is hard to describe their appearance, but we will liken them to a star-fish. The arm like processes, or psudo-pods, as they are termed, branch from all sides of the cell, and, when approaching a bacterium, those nearest the microbe extend out on either side, surround and envelope it. Thus the germ is evacuolated, and then digested by the phagocyte. Should bacteria be able to resist the attack of these phagocytes, or devouring cells, still they meet opposition. The blood serum is highly antiseptic, and thus has microbe-killing properties, and the oxygen taken into the circulation is fatal to many species of bacteria, as carbonic acid is to others. But these phagocytes are not confined to the blood vessels; they have the power to migrate from one tissue to another, and their usefulness is thus enhanced. The medical term for this migratory process is "diapedesis." (Of course it is necessary for physicians always to talk Greek).

Now, this is the way the human body in a state of good health, defends itself against bacterial attacks, but let the vitality of the system become lowered, through debility or other causes, and it looses some of its resistive power. It is in this abnormal state of the constitution that disease producing bacteria find it possible to develop within the organism, and cause disease by multiplying and excreting toxines that poison not only the tissues on which the bacteria grow, but distant portions of the body, for these poisonous alkaloids are taken into the circulation and are carried throughout the system. Phagocytes are quite susceptible to the action of bacterial toxines, and are weakened or killed by them.

Sometimes a bruise or cut will bring about serious results. Suppose a finger or hand is cut or pricked with a knife, pin, excavator, or any sharp instrument on which there are disease-producing bacteria. These germs are carried into the tissues, and inflammation results. The tissues become abnormal, and the bacteria, not meeting the natural resistance, are able to multiply. Phagocytes hurry to the place of irritation, and a battle ensues. The microbes multiply rapidly, and their excreted toxines act on the phagocytes, killing some and weakening others. The phagocytes pass through the vessel wall, and the bodies of those killed go to form pus. If the bacteria can resist the attack of phagocytes, and so vitiate the tissues as to be able to grow and multiply there, their excreted toxines gradually act on the surrounding parts, and if not checked or limited in their action, they cause blood-poisoning.

But where there is perfect health, disease will not occur, and as fresh air, plenty of exercise, frequent bathing, regularity in eating and sleeping, are all essential to good health, we can ill afford to neglect these important duties. Just so long as we observe the laws of health, we are doing our best to keep out disease.—*Ohio Dental Journal.*

